# Polygenic risk scores indicate extreme ages at onset of breast cancer in female *BRCA1/2* pathogenic variant carriers

**DOI:** 10.1186/s12885-022-09780-1

**Published:** 2022-06-27

**Authors:** Julika Borde, Yael Laitman, Britta Blümcke, Dieter Niederacher, Konstantin Weber-Lassalle, Christian Sutter, Andreas Rump, Norbert Arnold, Shan Wang-Gohrke, Judit Horváth, Andrea Gehrig, Gunnar Schmidt, Véronique Dutrannoy, Juliane Ramser, Julia Hentschel, Alfons Meindl, Christopher Schroeder, Barbara Wappenschmidt, Christoph Engel, Karoline Kuchenbaecker, Rita K. Schmutzler, Eitan Friedman, Eric Hahnen, Corinna Ernst

**Affiliations:** 1 Center for Familial Breast and Ovarian Cancer, Center for Integrated Oncology (CIO), Medical Faculty, University Hospital Cologne, Kerpener Straße 62, Cologne, 50937 Germany; 2 Oncogenetics Unit, Sheba Medical Center, Tel Hashomer, Israel; 3 Sackler School of Medicine, Tel Aviv University, Tel-Aviv, Israel; 4 Department of Gynaecology and Obstetrics, University Hospital Duesseldorf, Heinrich-Heine University, Duesseldorf, Germany; 5 Institute of Human Genetics, University of Heidelberg, Heidelberg, Germany; 6 Institute of Clinical Genetics, Technische Universitaet Dresden, Dresden, Germany; 7 Institute of Clinical Molecular Biology, Department of Gynaecology and Obstetrics, University Hospital of Schleswig-Holstein, Campus Kiel, Christian-Albrechts University Kiel, Kiel, Germany; 8 Department of Gynaecology and Obstetrics, University Hospital Ulm, Ulm, Germany; 9 Institute for Human Genetics, University Hospital Muenster, Muenster, Germany; 10 Institute of Human Genetics, Julius-Maximilians University, Wuerzburg, Germany; 11 Institute of Human Genetics, Hannover Medical School, Hannover, Germany; 12 Institute of Medical and Human Genetics, Charité Universitaetsmedizin, Berlin, Germany; 13 Department for Gynaecology and Obstetrics, Division of Tumor Genetics, Klinikum rechts der Isar, Technical University Munich, Munich, Germany; 14 Institute of Human Genetics, University of Leipzig Hospitals and Clinics, Leipzig, Germany; 15 Department of Gynaecology and Obstetrics, LMU Munich, University Hospital Munich, Munich, Germany; 16 Institute of Medical Genetics and Applied Genomics, University of Tübingen, Tübingen, Germany; 17 Institute for Medical Informatics, Statistics and Epidemiology (IMISE), Leipzig, Germany; 18 Division of Psychiatry, University College London, London, UK; 19 UCL Genetics Institute, University College London, London, UK

**Keywords:** Breast cancer, Polygenic risk score, PRS, Risk assessment, *BRCA1*, *BRCA2*

## Abstract

**Background:**

Clinical management of women carrying a germline pathogenic variant (PV) in the *BRCA1/2* genes demands for accurate age-dependent estimators of breast cancer (BC) risks, which were found to be affected by a variety of intrinsic and extrinsic factors. Here we assess the contribution of polygenic risk scores (PRSs) to the occurrence of extreme phenotypes with respect to age at onset, namely, primary BC diagnosis before the age of 35 years (early diagnosis, ED) and cancer-free survival until the age of 60 years (late/no diagnosis, LD) in female *BRCA1/2* PV carriers.

**Methods:**

Overall, estrogen receptor (ER)-positive, and ER-negative BC PRSs as developed by Kuchenbaecker et al. for BC risk discrimination in female *BRCA1/2* PV carriers were employed for PRS computation in a curated sample of 295 women of European descent carrying PVs in the *BRCA1* (n=183) or the *BRCA2* gene (n=112), and did either fulfill the ED criteria (n=162, mean age at diagnosis: 28.3 years, range: 20 to 34 years) or the LD criteria (n=133). Binomial logistic regression was applied to assess the association of standardized PRSs with either ED or LD under adjustment for patient recruitment criteria for germline testing and localization of *BRCA1/2* PVs in the corresponding BC or ovarian cancer (OC) cluster regions.

**Results:**

For *BRCA1* PV carriers, the standardized overall BC PRS displayed the strongest association with ED (odds ratio (OR) = 1.62; 95% confidence interval (CI): 1.16–2.31, *p*<0.01). Additionally, statistically significant associations of selection for the patient recruitment criteria for germline testing and localization of pathogenic PVs outside the *BRCA1* OC cluster region with ED were observed. For *BRCA2* PV carriers, the standardized PRS for ER-negative BC displayed the strongest association (OR = 2.27, 95% CI: 1.45–3.78, *p*<0.001).

**Conclusions:**

PRSs contribute to the development of extreme phenotypes of female *BRCA1/2* PV carriers with respect to age at primary BC diagnosis. Construction of optimized PRS SNP sets for BC risk stratification in *BRCA1/2* PV carriers should be the task of future studies with larger, well-defined study samples. Furthermore, our results provide further evidence, that localization of PVs in BC/OC cluster regions might be considered in BC risk calculations for unaffected *BRCA1/2* PV carriers.

**Supplementary Information:**

The online version contains supplementary material available at (10.1186/s12885-022-09780-1).

## Background

Inherited pathogenic variants (PVs) in the *BRCA1/2* genes are the most common cause of hereditary breast cancer (BC). Associated lifetime risks for BC development were assessed in a variety of studies, recent estimates range from 60 to 75% for female *BRCA1* and from 55 to 76% for female *BRCA2* germline PV carriers [1–4]. Clinical management of individuals found to be at high risk for BC focuses on risk reduction and early diagnosis of cancer, and includes early age (25–30 years) BC screening via magnetic resonance imaging (MRI) and mammograms, or risk-reducing mastectomy. Preventive medical treatment, e.g., with tamoxifen or denosumab, in unaffected *BRCA1/2* PV carriers is under discussion [5]. Accurate age-dependent estimates of BC penetrance in PV carriers are a crucial prerequisite in genetic counseling for making informed, individualized decisions about whether and when the risks associated with considered preventive measures are in relation to the expected BC risk.

BC incidence of *BRCA1* PV carriers rises sharply in the 4th decade of life and then remains at a similar, constant level [2]. The majority of tumors in *BRCA1* PV carriers are estrogen receptor-negative (ER-), but their proportion decreases with age [6, 7]. In *BRCA2* PV carriers, BC incidence increases rapidly until ages of 40 to 50 years, and then remains almost constant [2]. The majority of tumors are ER-positive (ER+), but the proportion of ER- tumors increases with age [6, 7].

BC risks of female *BRCA1/2* PV carriers were found to be affected by a variety of intrinsic and extrinsic factors, such as mammographic density [8, 9], family history of BC and ovarian cancer (OC), variant localization [2], birth cohort [10–12], reproductive history (e.g., age at menarche, first birth, and menopause, number of full-term pregnancies, breastfeeding) [13] and further modifiable factors such as body weight, physical activity, and alcohol consumption [14], among others.

Furthermore, numerous studies demonstrated that the effects of BC susceptibility loci, i.e., common single nucleotide polymorphisms (SNPs), which individually contribute only slightly to individual BC risks, but whose effects can be summed up to polygenic risk scores (PRSs) are able to achieve a clinically relevant degree of BC risk discrimination [8, 15–20]. However, it was also consistently shown that PRS risk stratification is reduced in *BRCA1/2* PV carriers in comparison to the general population [21–23]. Moreover, a recent case-only study by Coignard et al. [24] comprising more than 60,000 unselected BC cases and 13,000 cases with *BRCA1/2* mutations, provided evidence that several SNPs associated with BC risk in the general population, and are therefore considered in PRS calculations, are in fact associated with the *BRCA1/2* mutation status, and hence do not have effects on BC risk in *BRCA1/2* PV carriers.

In order to construct a highly informative study sample with respect to modifying factors of BC risk in female *BRCA1* PV carriers, Sepahi et al. introduced the approach of investigating patients with “extreme phenotypes” due to age at primary BC diagnosis [25]. The authors analyzed a sample of 133 *BRCA1*-positive patients who were either diagnosed with primary BC at an age younger than 35 years (early diagnosis, ED) or remained cancer-free until the age of 60 years (late/no diagnosis, LD). The study concluded with the assumption that co-occurring truncating variants in further DNA repair genes associate with ED, although no statistical significance was reached to prove this hypothesis. All patients examined in the study of Sepahi et al. met the inclusion criteria of the German Consortium for Hereditary Breast and Ovarian Cancer (GC-HBOC) for germline testing (Additional file 1: Table S1), i.e. were highly selected for BC/OC family history and/or early BC/OC onset.

Here, we revisit the approach of Sepahi et al. to assess the utility of PRSs for the discrimination of extreme phenotypes with respect to age at BC onset in female *BRCA1/2* PV carriers. We employed SNP sets for PRS computation as developed and evaluated by Kuchenbaecker et al. [22], which to our knowledge are the only published PRS SNP sets to date specifically adapted for BC risk stratification in women with a *BRCA1/2* PV, namely an overall BC PRS comprising 88 loci, a PRS specific for ER+ BC comprising 87 SNPs and a PRS specific for ER- BC comprising 53 SNPs. In contrast to Sepahi et al., our sample is not only composed of patients selected by the GC-HBOC inclusion criteria for germline testing, but also by *BRCA1/2* PV carriers recruited at the Suzanne Levy-Gertner Oncogenetics Unit at the Sheba Medical Center (Tel Aviv University, Israel). Due to a less restrictive access to an initial screening for Israeli *BRCA1/2* founder mutations [26], these individuals are expected to be unselected for family history and/or early BC/OC onset.

## Methods

### Study sample

As a PV, we defined class 4/5 protein-truncating variants with respect to 5-tier variant classification system suggested by the International Agency for Research on Cancer (IARC). Variant classification was performed according to the guidelines published by the Evidence-based Network for the Interpretation of Germline Mutant Alleles (ENIGMA) [27] and the American College of Medical Genetics and Genomics (ACMG) [28]. Only female *BRCA1/2* PV carriers with a primary BC diagnosis before the age of 35 years (ED group) or cancer-free survival until the age of 60 years (LD group) were considered. No individual in the ED group was diagnosed with OC prior to BC, and no individual in the LD group underwent prophylactic mastectomy before the age of 60 years.

All patients in the study sample were identified through diagnostic germline testing and recruited between 1996 and 2017 by 13 centers of the GC-HBOC and at Suzanne Levy-Gertner Oncogenetics Unit at the Sheba Medical Center (Tel Aviv University). Individuals recruited by the GC-HBOC were selected by the corresponding inclusion criteria for germline testing (Additional file 1: Table S1), whereas the *BRCA1/2* PV carriers recruited by the Sheba Medical Center met the broader Israeli inclusion criteria for *BRCA1/2* screening [26], and are therefore considered unselected. All patients were tested for PVs in *BRCA1/2* and the *CHEK2*:c.1100delC variant. Individuals with PVs both in *BRCA1* and *BRCA2* (n=1) and individuals with co-occurring variant *CHEK2*:c.1100delC (n=1) were excluded.

Regarding the classification of variant localizations within *BRCA1/2*, we applied – in concordance with Sepahi et al. [25] – the definitions of Rebbeck et al. [29], i.e., *BRCA1* variants intersecting with regions c.179–505, c.4328–4945 or c.5261–5563 (with respect to transcript NM_007294.4) were considered to be localized within a breast cancer cluster region (BCCR) and variants intersecting with regions c.1380–4062 were considered to be localized within an ovarian cancer cluster region (OCCR). For *BRCA2*, PVs intersecting with c.1–596, c.772–1806 or c.7394–8904 (with respect to transcript NM_000059.4) were evaluated as being localized within a BCCR, and PVs intersecting with c.3249–5681 or c.6645-7471 as being localized within an OCCR.

### SNP genotyping

For SNP genotyping, we used a customized 48.48 amplicon-based target enrichment panel (Access Array®, Fluidigm, San Francisco, CA, USA). Variants which could not be covered due to technical limitations, were replaced by adjacent SNPs in linkage disequilibrium (Additional file 2: Table S2). Subsequent parallel next-generation sequencing (NGS) of the barcoded amplicons of the samples was performed by employing an Illumina NextSeq500 sequencing device (Illumina, San Diego, CA, USA) as described previously [17]. All DNA samples were centrally analyzed at the Center for Familial Breast and Ovarian Cancer, University Hospital Cologne, Germany. Raw BCL files were demultiplexed using bcl2fastq2 Conversion Software v2.19 available at https://support.illumina.com Sequence reads were mapped to the human reference genome assembly GRCh37, including decoy sequences (hs37d5), using BWA-MEM of Burrows-Wheeler Aligner (BWA) v0.7.15 [30], and target-specific primer sequences were removed using BAMClipper v1.1 [31]. SNP calling was performed on a merged BAM file including all samples using FreeBayes v1.0.0 [32] under specification of common SNPs from dbSNP build 151 [33] as variant input, and under specification of -q 20, i.e., a minimum phred-scaled base quality of 20. Variant calls were filtered for a minimum read depth of 10 and converted to TPED format using PLINK v1.9 [34].

### Quality control & PRS computation

Quality controls were performed as previously described [17]. SNP calls and samples were filtered for a minimum call rate of 0.95. One sample was identified as an outlier due to heterogeneity using the Bonferroni-corrected upper and lower 5 percentiles of the empricially estimated normal distribution and excluded. Samples with putative African or putative Asian ancestry were identified by multi-dimensional scaling based on a combined set of 342 overlapping SNPs in the sample and 1000 Genomes data. Four individuals were excluded because African or Asian ancestry could not be ruled out (Additional file 3: Figure S1).

For each person *i*, an indvidual PRS was derived via

$$\text{PRS}_{i} = \sum\limits_{j=1}^{N} \beta_{j} g_{ij}\ \text{with}\ g \in \{0,1,2\}, $$

where *β* is the per-allele log odds ratio and *g*_*ij*_ is the number of effect alleles in person *i* for locus *j*. Missing genotypes were imputed to the average observed genotype in the sample. Alleles with complementary alleles, i.e., either C/G or A/T, were excluded from PRS calculation due to ambiguity. This criterion also applied to SNP rs11571833, which is located within *BRCA2*. Quality filters resulted in effective SNP set sizes of *N*=77 for the overall and ER+ BC PRS, and of *N*=50 for the ER- BC PRS (Additional file 4: Table S3). Of the total 86 SNPs employed for PRS calculations, four showed statistically significant differences in observed allele frequencies when comparing samples recruited by GC-HBOC and by Sheba Medical Center (Bonferroni-corrected Fisher’s exact test *p*<0.05, Additional file 4: Table S3). PRSs for each SNP set were standardized to have mean 0 and variance 1.

### Statistical analysis

Analyses were conducted using the GenABEL v1.8 utilities [35], R v3.6 and PLINK v1.9 [34]. All statistical tests were two-sided with *p* values below 0.05 considered statistically significant. The association of standardized PRSs with ED, respectively LD, of primary BC was assessed by employing a binomial logistic regression model (outcome 1: ED, outcome 0: LD) under adjustment for selection by the GC-HBOC inclusion criteria for germline testing and localization of *BRCA1/2* PVs within the corresponding BCCRs and OCCRs.

A robust sandwich variance estimation approach [36], clustering observations based on family ID, was applied to account for related individuals in the sample.

## Results

Quality controls resulted in a curated sample set comprising 295 *BRCA1/2* PV carriers out of 293 families as input for the PRS computation (183 *BRCA1*; 112 *BRCA2*, Additional file 5: Table S4)). Of the total 295 *BRCA1/2* PV carriers, 162 individuals developed BC before the age of 35 years and therefore belonged to the ED group. From the 133 individuals in the LD group (cancer-free survival until the age of 60 years), 41 were known to be diagnosed with BC after the age of 60 years. Key characteristics of the study sample are given in Table 1.

**Table 1:** Key characteristics of the study sample. BC: breast cancer; ER: estrogen receptor; OC: ovarian cancer; PV: pathogenic variant; (not) sICGT: (not) selected by the inclusion criteria for germline testing of the German Consortium for Hereditary Breast and Ovarian Cancer. *Diagnosis after age 60

	total	ED	LD
study sample	295	162	133
with BC	203	162	41*
sICGT	188	114	74
≥1 relative with BC/OC	170	99	71
not sICGT	107	48	59
*BRCA1* PV carriers	183	116	67
PV within BCCR		33	19
PV within OCCR		21	19
*BRCA2* PV carriers	112	46	66
PV within BCCR		12	7
PV within OCCR		16	14
ER status known, first BC	136	105	31
ER+	62	44	18
ER-	74	61	13
ER status unknown	67	57	10

**Fig. 1:**
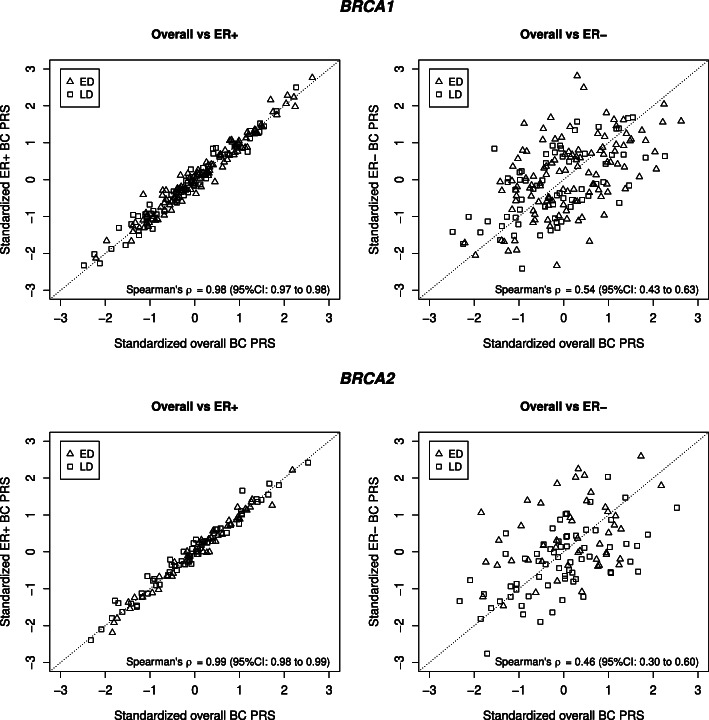
Standardized polygenic risk score (PRS) values per individual and Spearman’s rank correlation coefficients *ρ*. (Upper left) Overall breast cancer (BC) PRS versus estrogen receptor-positive (ER+) BC PRS in 183 *BRCA1* pathogenic variant (PV) carriers who were either diagnosed with BC before the age of 35 years (early diagnosis, ED) or were unaffected at least until the age of 60 years (late/no diagnosis, LD). (Upper right) Overall BC PRS versus estrogen receptor-negative (ER-) BC PRS in the same sample. (Lower left) Overall BC PRS versus ER+ BC PRS in 112 *BRCA2* PV carriers who belonged to either the LD or ED group. (Lower right) Overall BC PRS versus ER- BC PRS in the same sample

Standardized overall BC and ER+ BC PRSs showed a strong correlation at the individual level (*BRCA1* PV carriers: Spearman’s correlation coefficient *ρ*=0.98 (95% confidence interval (CI): 0.97–0.98); *BRCA2* PV carriers: *ρ*=0.99 (95% CI: 0.98–0.99), Fig. 1). Correlations between the individual standardized overall BC and ER- BC PRSs were considerably lower in comparison, but also statistically significant (*BRCA1* PV carriers: *ρ*=0.54 (95% CI: 0.43–0.63); *BRCA2* PV carriers: *ρ*=0.46 (95% CI: 0.30–0.60), Fig. 1).

**Fig. 2:**
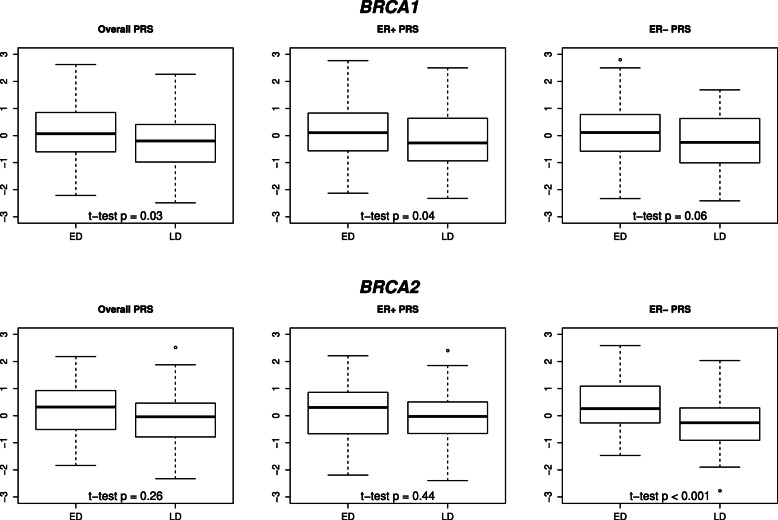
Boxplots and two-sided Welch’s t-test *p* values of standardized polygenic risk scores (overall breast cancer (BC), estrogen receptor (ER)-positive BC (ER+), ER-negative BC (ER-) PRS) of 183 *BRCA1* and 112 *BRCA2* pathogenic variant carriers, who were either diagnosed with BC before the age of 35 years (early diagnosis, ED) or were unaffected at least until the age of 60 years (late/no diagnosis, LD)

For both *BRCA1* and *BRCA2* PV carriers, and consistently across all PRSs under consideration, the average PRS was increased in the ED group in comparison to the average PRS in the LD group (Fig. 2). However, these differences reached statistical significance exclusively considering the overall or ER+ BC PRS of the *BRCA1* PV carriers (two-sided Welch’s t-test *p*≤0.04), and the ER- BC PRS of the *BRCA2* PV carriers in our study sample (two-sided Welch’s t-test *p*<0.001).

Binomial logistic regression analyses were employed to assess the association of the standardized PRSs with ED of BC taking selection by the GC-HBOC inclusion criteria and localization of *BRCA1/2* PVs, i.e., localization within the BCCRs or OCCRs, into account.

For *BRCA1* PV carriers, among the three different PRS SNP sets employed, the standardized overall BC PRS displayed the strongest association with ED (odds ratio (OR) = 1.62; 95% CI: 1.16–2.31, *p*=0.007, Table 2) in our analysis. Additionally, a statistically significant association between selection by the GC-HBOC inclusion criteria for germline testing and ED with ORs ranging from 2.84 to 3.52 (*p*≤0.005) was observed. Localization of PVs within the *BRCA1* OCCR was statistically significantly associated with LD with ORs ranging from 0.32 to 0.35 (*p*≤0.02), whereas no statistically significant effect of localization of PVs within the *BRCA1* BCCRs was observed.

**Table 2:** Binomial logistic regression analysis results. Odds ratios (ORs), 95% confidence intervals (CIs) and *p* values based on two-tailed z-test for binomial logisitic regression analyses with extremely young age (<35 years) at breast cancer (BC) diagnosis (early diagnosis, ED) as the output considering standardized polygenic risk scores (PRSs), selection by the inclusion criteria for germline testing of the German Consortium for Hereditary Breast and Ovarian Cancer (sICGT) and localization of pathogenic variants (PVs) within the corresponding BC cluster regions (BCCRs) or OC cluster regions (OCCRs). Three PRS SNP sets were employed for PRS computation: overall BC, estrogen-receptor-positive (ER+) BC and ER-negative (ER-) BC PRS [22]. 183 *BRCA1* and 112 *BRCA2* PV carriers were considered who were either diagnosed with BC before the age of 35 years (ED) or unaffected until the age of 60 years

	OR	95%CI	*p*
*BRCA1* PV carriers			
overall BC PRS	1.62	1.16–2.31	0.007
inside BCCR	0.65	0.30–1.39	0.26
inside OCCR	0.32	0.13–0.75	0.01
sICGT	3.45	1.67–7.43	0.002
ER+ BC PRS	1.60	1.14–2.30	0.01
inside BCCR	0.65	0.30–1.39	0.26
inside OCCR	0.32	0.13–0.75	0.01
sICGT	3.52	1.70–7.63	0.002
ER- BC PRS	1.38	1.00–1.92	0.05
inside BCCR	0.62	0.29–1.33	0.16
inside OCCR	0.35	0.15–0.81	0.02
sICGT	2.84	1.42–5.89	0.005
*BRCA2* PV carriers			
overall BC PRS	1.30	0.87–1.96	0.21
inside BCCR	2.94	0.96–9.55	0.06
inside OCCR	2.02	0.75–5.64	0.16
sICGT	2.21	0.77–6.80	0.15
ER+ BC PRS	1.21	0.81–1.82	0.37
inside BCCR	2.95	0.97–9.55	0.06
inside OCCR	1.98	0.74–5.49	0.17
sICGT	2.20	0.77–6.75	0.15
ER- BC PRS	2.27	1.45–3.78	<0.001
inside BCCR	2.77	0.87–9.38	0.07
inside OCCR	1.88	0.66–5.56	0.25
sICGT	2.32	0.77–7.48	0.13

Considering *BRCA2* PV carriers, the strongest association with ED was observed for the ER- BC PRS (OR = 2.27; 95% CI: 1.45–3.78, *p*<0.001), whereas the overall and ER+ BC PRS did not show statistically significant association with ED (Table 2). Regardless of the PRS SNP set employed, no statistically signifcant effect of selection by the GC-HBOC inclusion criteria for germline testing and localization of PVs within the *BRCA2* OCCRs or BCCRs was observed.

## Discussion

In our study considering female *BRCA1/2* PV carriers who either developed BC before the age of 35 years (ED) or remained cancer-free until the age of 60 years (LD), we assessed whether extreme age of BC onset due to the definition of Sepahi et al. [25] can be explained by PRSs, among other known factors. Three subtype-specific PRS SNP sets as proposed by Kuchenbaecker et al. [22] for BC risk discrimination in *BRCA1/2* PV carriers were employed, namely an overall BC, an ER+ BC and an ER- BC PRS. All PRS SNP sets investigated consist of a maximum of 88 loci and therefore have the potential to be straightforwardly implemented in routine diagnostic multi-gene panel analyses.

Our findings indicate the highest ability of ED/LD discrimination in *BRCA1* PV carriers if applying the overall BC PRS, and a slightly reduced but comparable performance of the ER+ PRS. For *BRCA2* PV carriers, the ER- BC PRS showed the highest ability of ED/LD discrimination. The observed associations were statistically significant, allowing us to clearly demonstrate the contribution of PRSs to the development of extreme phenotypes of *BRCA1/2* PV carriers with respect to age at primary BC diagnosis.

However, previous studies applying Cox regressions with years of life until first BC diagnosis as the outcome, reported the highest potential for BC risk stratification of the ER- BC PRS for *BRCA1* PV carriers and of the overall BC PRS for *BRCA2* PV carriers [22, 37]. Kuchenbaecker et al. analyzed data for 15,252 *BRCA1* and 8,211 *BRCA2* PV carriers from the Consortium of Investigators of Modifiers of *BRCA1/2* (CIMBA) to develop and evaluate the PRS SNP sets employed here [22]. It is remarkable, that the authors focused primarily on distinguishing between carriers and noncarriers of a *BRCA1/2* PV (which is essentially equivalent to distinguishing between carriers and noncarriers of a pathogenic alteration in a BC risk gene with high penetrance), but not between *BRCA1* and *BRCA2* PV carriers per se. Separate evaluation, however, concluded that the ER- BC PRS performed best for *BRCA1* PV carriers, whereas for *BRCA2* PV carriers, the overall BC PRS showed the strongest association. This conclusion was confirmed by Barnes et al. [37], who retrospectively evaluated the overall, ER+ and ER- version of a 313 PRS SNP set adapted to BC risk stratification in women unselected for germline mutation status [16]. This study was also performed on the basis of CIMBA data, and involved almost 19,000 female *BRCA1* and more than 12,000 female *BRCA2* PV carriers. Besides the different outcomes considered in regression analyses, the deviating conclusions regarding the best-performing PRSs could be due to artificial enrichment with LD cases in our study design. Only 6.1% (928/15,252) of the *BRCA1* and 10.1% (833/8,211) of the *BRCA2* PV carriers examined by Kuchenbaecker et al. did not receive a primary BC diagnosis until after an age of 60 years or were known to have no disease by that age, and thus, meeting the LD criteria; in the study by Barnes et al. this was true for 6.0% (1143/18,935) and 10.1% (1251/12,339), respectively.

Further, in our sample the proportion of the ER+ subtype among all BC cases of *BRCA1* PV carriers in the ED group was 29.4% (20/68) and 55.6% (5/9) in the LD group, with missing data of receptor status for 52 tumors (ED: 48, LD: 4), whereas Mavaddat et al. reported in agreement with previous findings by Foulkes et al. an ER+ proportion of <20% among BC diagnoses before age 40 years, and of approximately 45% and >50% among BC diagnoses between ages 60 and 69 years, and >70 years, respectively [6, 7]. Regarding *BRCA2* PV carriers in our sample, the ER+ proportion was 64.9% (24/37) in the ED group, with no data available for 9 tumors, and 59.1% (13/22) in the LD group, with missing data for 6 tumors. In contrast, Mavaddat found a proportion of >80% of ER+ tumors among BC diagnoses between the ages of 30 and 39 years in *BRCA2* PV carriers, and of >70%, respectively >60%, for BC diagnoses between 60 and 69 years, respectively >70 years, of age [7]. Thus, the proportion of ER- tumors was lower than expected for *BRCA1* PV carriers and higher as expected for *BRCA2* PV carriers in our sample, which may also have led to divergent results regarding the comparison of performances between PRS sets compared with previous studies based on CIMBA data.

Our results, based on a sample independent of previous genome-wide association studies (GWASs) for PRS construction, also highlight the urgent need for a careful review of BC PRSs specific to *BRCA1/2* PV carriers and their performance. The question is not only whether an ER-specific or overall BC PRS should be used, but also whether the PRS is equally appropriate for risk stratification in young and older women and whether it is even justified to use a common PRS for *BRCA1* and *BRCA2* PV carriers. Coignard et al. [24] suggested that several SNPs included in PRS computation are associated with *BRCA1/2* mutation status rather than with BC risk in general. The authors presented 7 SNPs associated with *BRCA1* carrier status and 3 SNPs associated with *BRCA2* carrier status, of which 7 (*BRCA1*-associated: rs13281615, rs704010, rs4784227, rs616488 and rs17530068; *BRCA2*: rs10759243 and rs6001930) were identical or in linkage disequilibrium to SNPs included in the PRS calculations presented. In addition, Coignard et al. identified two, respectively three, loci that modify BC risks in *BRCA1*, respectively *BRCA2*, mutation carriers exclusively, which point towards the need for establishment of divergent PRSs for *BRCA1* and *BRCA2* PV carriers. However, our sample was too small to contribute to these efforts by investigating the associations of individual SNPs with BC with sufficient statistical power.

The study of Sepahi et al. [25] considering 133 *BRCA1* PV carriers fulfilling either the ED or LD criteria, pointed towards an association of germline *BRCA1* PVs within the OCCR with LD, although no statistical significance was reached. Our study now shows a statistically significant association based on a sample of 183 *BRCA1* PV carriers. Consistent with Sepahi et al., there was no statistically significant effect of localization of PVs within the *BRCA1* BCCRs. With regard to the localization of PVs in the *BRCA2* gene, no statistically significant associations with ED were found, but this is probably due to the small sample size of only 112 PV carriers.

Besides limited sample size, this study has further limitations, as co-occuring truncating variants in DNA-repair genes were not considered as suggested for discrimination between extreme phenotypes with respect to age at BC onset by Sepahi et al. [25] and possible biases due to patient recruitment in two different countries can not be excluded. Further, adjustment of analyses for year of birth was not feasible, as considered individuals from the ED group were born in 1953 at the earliest, and individuals from the LD group no later than 1957.

## Conclusions

Although the optimal PRS SNP set for this purpose remains to be found in future studies with larger sample size, our results show that the PRS is an essential prerequisite for the clinical management of unaffected *BRCA1/2* PV carriers, as it helps to identify those women who should be offered prophylactic measures at a comparatively very young age. Furthermore, our results provide further evidence that the localization of pathogenic *BRCA1/2* mutations should be considered in BC risk calculations.

## Supplementary Information

**Additional file 1** Inclusion criteria of the German Consortium for Hereditary Breast and Ovarian Cancer (GC-HBOC) for germline testing.

**Additional file 2** SNPs replaced by SNPs in linkage disequlibrium.

**Additional file 3** First three dimensions of multi-dimensional scaling of a combined set of the curated study sample and 2,157 individuals of known ancestry, namely European (EUR), African (AFR), East Asian (EAS), or South Asian (SAS), from the 1000 Genomes data comprising 342 SNPs. Samples that were identified as of putative African or Asian origin from the study sample are shown in red (ExPh OUT).

**Additional file 4** SNPs included in polygenic risk score (PRS) computation, corresponding per-allele log odds ratios, and comparison of allele frequencies between patients recruited by Sheba Medical Center, Tel Aviv University (AF Sheba) and patients recruited by the German Consortium for Hereditary Breast and Ovarian Cancer (AF GC-HBOC).

**Additional file 5** Prevalence of IARC class 4/5 pathogenic germline variants in the *BRCA1* gene (considering transcript NM_007294.4) and the *BRCA2* gene (considering transcript NM_000059.4) in the curated final study sample consisting of 295 individuals.

## Data Availability

The dataset produced or analyzed in this study is not publicly available due to privacy reasons but it will be available from the corresponding author upon reasonable request.

## Abbreviations

BC: Breast cancer
BCCR: Breast cancer cluster region
*BRCA1*: *Breast cancer 1* gene
*BRCA2*: *Breast cancer 2* gene
CI: Confidence interval
ED: Early diagnosis
ER+: Estrogen receptor-positive
ER-: Estrogen receptor-negative
GC-HBOC: German Consortium for Hereditary Breast and Ovarian Cancer
LD: Late diagnosis
OC: Ovarian cancer
OCCR: Ovarian cancer cluster region
OR: Odds ratio
PRS: Polygenic risk score
PV: Pathogenic variant
SNP: Single nucleotide polymorphism
