# Supplementary material for: Polygenic risk scores indicate extreme ages at onset of breast cancer in female BRCA1/2 pathogenic variant carriers
Source: BMC Cancer. 2022 Jun 27;22:706. doi: 10.1186/s12885-022-09780-1 (PMC9238030; doi:10.1186/s12885-022-09780-1)
Supplement: Supplementary file 1 — Additional file 1 Inclusion criteria of the German Consortium for Hereditary Breast and Ovarian Cancer (GC-HBOC) for germline testing. [file 12885_2022_9780_MOESM1_ESM.docx]

- ≥3 women with breast cancer
- ≥2 women with breast cancer, 1 with onset below 51 years of age
- ≥1 woman with breast cancer and 1 woman with ovarian cancer
- ≥2 women with ovarian cancer
- ≥1 woman with breast and ovarian cancer
- ≥1 woman with breast cancer below 36 years of age
- ≥1 woman with bilateral breast cancer with onset below 51 years
- ≥1 male with breast cancer and 1 woman with breast or ovarian cancer
