# Supplementary figures and images for: Polygenic risk scores indicate extreme ages at onset of breast cancer in female BRCA1/2 pathogenic variant carriers
Source: BMC Cancer. 2022 Jun 27;22:706. doi: 10.1186/s12885-022-09780-1 (PMC9238030; doi:10.1186/s12885-022-09780-1)

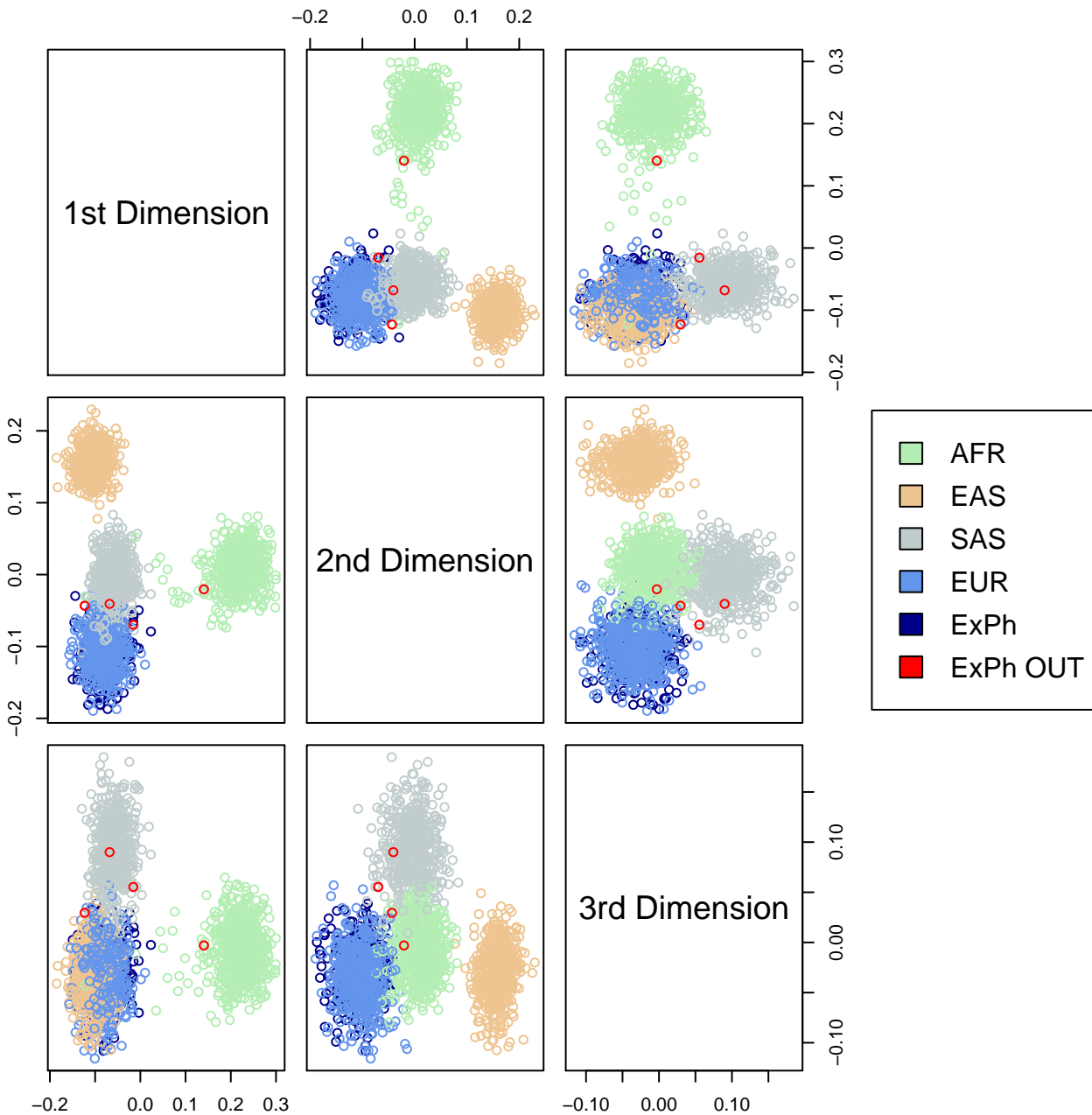

Supplement: Supplementary file 3 — Additional file 3 First three dimensions of multi-dimensional scaling of a combined set of the curated study sample and 2,157 individuals of known ancestry, namely European (EUR), African (AFR), East Asian (EAS), or South Asian (SAS), from the 1000 Genomes data comprising 342 SNPs. Samples that were identified as of putative African or Asian origin from the study sample are shown in red (ExPh OUT). [file 12885_2022_9780_MOESM3_ESM.pdf]
